# Clinical outcomes of perforator-based propeller flaps versus free flaps in soft tissue reconstruction for lower leg and foot trauma: a retrospective single-centre comparative study

**DOI:** 10.1186/s12891-024-07433-x

**Published:** 2024-04-16

**Authors:** Mitsutoshi Ota, Makoto Motomiya, Naoya Watanabe, Kohei Shimoda, Norimasa Iwasaki

**Affiliations:** 1 Department of Orthopaedic Surgery, Obihiro Kosei Hospital Hand Center, Nishi 14 Minami 10, Obihiro, 080-0024 Japan; 2 Department of Orthopaedic Surgery, Faculty of Medicine and Graduate School of Medicine, Hokkaido University, Sapporo, Japan

**Keywords:** Trauma, Lower leg, Soft tissue reconstruction, Free flap, Perforator flap, Propeller flap

## Abstract

**Background:**

The efficacy and safety of perforator-based propeller flaps (PPF) versus free flaps (FF) in traumatic lower leg and foot reconstructions are debated. PPFs are perceived as simpler due to advantages like avoiding microsurgery, but concerns about complications, such as flap congestion and necrosis, persist. This study aimed to compare outcomes of PPF and FF in trauma-related distal lower extremity soft tissue reconstruction.

**Methods:**

We retrospectively studied 38 flaps in 33 patients who underwent lower leg and foot soft tissue reconstruction due to trauma at our hospital from 2015 until 2022. Flap-related outcomes and complications were compared between the PPF group (18 flaps in 15 patients) and the FF group (20 flaps in 18 patients). These included complete and partial flap necrosis, venous congestion, delayed osteomyelitis, and the coverage failure rate, defined as the need for secondary flaps due to flap necrosis.

**Results:**

The coverage failure rate was 22% in the PPF group and 5% in the FF group, with complete necrosis observed in 11% of the PPF group and 5% of the FF group, and partial necrosis in 39% of the PPF group and 10% of the FF group, indicating no significant difference between the two groups. However, venous congestion was significantly higher in 72% of the PPF group compared to 10% of the FF group. Four PPFs and one FF required FF reconstruction due to implant/fracture exposure from necrosis. Additionally, four PPFs developed delayed osteomyelitis post-healing, requiring reconstruction using free vascularized bone graft in three out of four cases.

**Conclusions:**

Flap necrosis in traumatic lower-leg defects can lead to reconstructive failure, exposing implants or fractures and potentially causing catastrophic outcomes like osteomyelitis, jeopardizing limb salvage. Surgeons should be cautious about deeming PPFs as straightforward and microsurgery-free procedures, given the increased complication rates compared to FFs in traumatic reconstruction.

**Data access statement:**

The datasets generated during and/or analyzed during the current study are available from the corresponding author on reasonable request.

## Introduction

Early soft tissue coverage is important for obtaining good function without complications when treating a severe open injury of the lower extremity [1, 2]. Although simple and reliable pedicled gastrocnemius muscle flaps can be used for the reconstruction of the proximal part of the lower leg, it is often difficult to reconstruct the soft tissue defect using a pedicled flap from the middle third of lower leg to the foot because of the thin soft tissue and poor blood circulation [3, 4]. Although various reconstruction procedures, such as the reverse sural arterial flap, perforator flap, and free flap (FF), have been reported for treating soft tissue defects from the middle third of the lower leg to the foot [5–8], the decision about the best procedure can be complicated by the risk that flap failure can lead to serious complications such as osteomyelitis and amputation when used for severe lower leg and foot injuries [9, 10].

FF is the primary option for soft tissue reconstruction of the distal leg and foot. The perforator-based propeller flap (PPF), as reported by Hyakusoku et al. [11], has attracted recent interest as a new procedure for lower leg reconstruction due to its advantages such as avoiding the need for microsurgery, providing like-with-like cosmetic reconstruction effects, and shorter operation times [5, 12–15]. However, flap congestion and partial necrosis at the tip of the flap are considered significant complications of PPF, and some surgeons have warned against its use for soft tissue defects caused by trauma [10, 16]. Studies on reconstruction using PPF are sporadic, but many studies have included flaps not only for trauma but also for reconstruction after tumor resection [12, 16–18]. Few comparative studies have reported on the use of PPF for reconstruction of trauma-related soft tissue defects in the lower leg and foot.

This study aimed to compare outcomes of PPF and FF in trauma-related distal lower extremity soft tissue reconstruction.

## Methods

This study was approved by the Institutional Review Board (IRB) at Obihiro Kosei Hospital (Approval No. 2023–05). We retrospectively reviewed cases in which reconstruction was performed for trauma-related soft tissue defects in the lower leg and foot at our institution between November 2015 and October 2022. We extracted series of cases in which soft tissue reconstruction was performed using PPF or FF from the soft tissue reconstruction database at our hospital. Of these, cases with open wounds reconstructed more than 6 weeks post-trauma, cases involving chronic osteomyelitis that developed more than 3 months after the initial injury, soft tissue defects in the toes, and pedicled flaps, such as the reverse sural arterial flap or gastrocnemius muscle flap, were excluded. After this exclusion, 38 flaps in 33 patients were extracted and classified into two groups according to the reconstruction procedure: the PPF group (18 flaps in 15 patients) and the FF group (20 flaps in 18 patients).

Data regarding background, comorbidities, and preoperative systemic conditions, classified according to the American Society of Anesthesiologists (ASA) classification system, were retrospectively collected from medical records. Information pertaining to soft tissue defects and flap details was obtained from surgical records, as well as intraoperative photos and videos. Defect sizes were categorized based on previous reports [16]. The presence or absence of calcification in the main vessels of the pedicle of the PPF and the recipient vessels of the FF was assessed using simple X-ray. Flap outcomes and postoperative complications were investigated in accordance with the Clavien–Dindo classification [19]. In addition to assessing complete and partial flap necrosis, we also investigated the coverage failure rate, defined as requiring secondary flaps for flap necrosis [20]. We classify any case requiring surgical interventions such as debridement, secondary closure, skin grafting, and secondary flaps as partial necrosis, irrespective of the extent of necrosis. Flap-related complications were classified separately as early (< 3 weeks) and delayed (> 3 weeks) stages [10]. All patients had been followed up for at least 3 months, and the final gait status as an indicator of patient mobility was investigated [21].

### Surgical technique

**Fig. 1:**
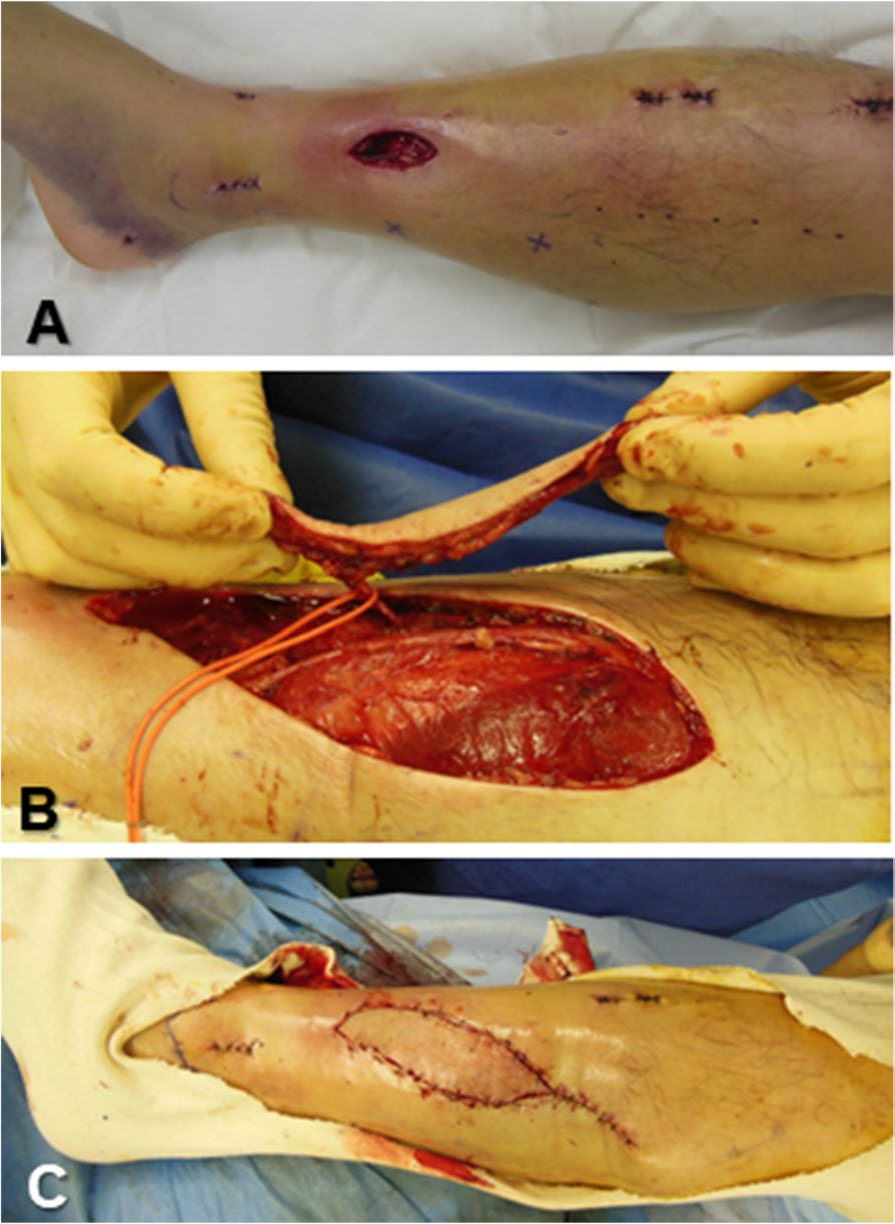
**A** An open tibial shaft fracture in a 55-year-old man. After osteosynthesis with an intramedullary nail, a 45 × 25 mm soft tissue defect was left just above the fracture. **B** Ten days after the injury, the perforator of the posterior tibial artery was dissected in a skeletonized state and the perforator-based propeller flap was elevated. **C** The flap was rotated 180 degrees, and the donor site was closed without a skin graft. Slight congestion was observed at the tip of the flap after surgery, but the flap survived without any problems

All surgeries were performed by 2 senior hand surgeons. For PPF, color Doppler ultrasound was used preoperatively to identify perforating branches near the defect and confirm their passage to the main arteries. The flap was designed to contain the perforator. First, the flap was raised subfascially and the perforator vessel was confirmed under the fascia and carefully dissected. After circumferentially dissecting the skin flap, the perforator was fully dissected toward the major artery. As reported by Soteropulos et al. [22], when the rotation angle was large, the perforator was skeletonized to prevent twisting of the vascular pedicle during flap rotation. We visually assessed the flap colour and capillary refilling to evaluate intraoperative blood flow. After transferring the flap to the defect without tension, the donor was closed using direct suturing or skin grafting (Fig. 1). In the PPF group, patients were confined to bed rest for three days following the flap operation. In instances where congestion of the skin flap occurred postoperatively, efforts were made to salvage the flap by removing stitches and/or utilizing medical leech therapy, and the duration of rest was prolonged as deemed necessary.

For FF, either a fasciocutaneous flap or myocutaneous flap was selected depending on the shape of the soft tissue defect. A major artery was selected as the recipient vessel, and anastomosis was performed in an end-to-end (ETE) or end-to-side (ETS) fashion. In early cases, the patients were administered prostaglandin E1 continuously (40–80 μg/day for 1 week) after surgery, but not in later cases. After surgery, the patients were restricted to bed rest for one week.

Smoking was prohibited for at least three weeks after surgery in both the PPF and FF groups. If the necrotic area of the skin flap was large and there were concerns about bone or implant exposure, a secondary flap was inserted early.

### Statistical analysis

The chi-squared test, Fisher’s exact test, unpaired *t* test, or Mann–Whitney *U* test was used to compare the two groups as appropriate. Statistical significance was set at *p* value < 0.05. Regarding Fisher's exact test, odds ratios (OR) and 95% confidence intervals (CI) were reported when deemed necessary. All analyses were performed using Bell Curve for Excel (version 3.21; Social Survey Research Information Co., Ltd., Osaka, Japan).

## Results

### Patient demographics and details of soft tissue defects

The two groups did not differ significantly in terms of patient background (sex, age, body mass index, complications, and ASA grading) (Table 1). In the background of soft tissue defects, the PPF group included more cases with the etiology of postoperative wound necrosis after initial osteosynthesis, with the defect location in the distal third of the lower leg and a smaller defect size. In the FF group, open fracture was the most frequent cause of larger soft tissue defects, and some cases included soft tissue defects of the foot. For the management of open wounds, most patients in the PPF group were referred midway through their treatment by a general orthopaedic surgeon, whereas most patients in the FF group were treated by the reconstructive team from the initial treatment onward. The time from the onset of the open wound to soft tissue reconstruction was significantly longer in the PPF group than in the FF group (Table 2).

**Table 1:** Patient demographics

		**PPF**	**FF**	***p*** ** value**
Numbers of patients		15	18	
Sex				0.45
	Male	9	14	
	Female	6	4	
Age (yr): mean ± SD (range)		58 ± 22 (21–95)	58 ± 17 (27–85)	0.96
BMI (kg/m^2^): mean ± SD (range)		24 ± 4 (18–36)	23 ± 4 (17–32)	0.86
Comorbidity		10	16	0.20
	DM	4	5	1.00
	PVD	0	1	1.00
	HT	5	5	1.00
	Haemodialysis	1	1	1.00
	Others	5	11	0.17
Smoking		4	7	0.47
ASA				0.14
	I	6	2	
	II	8	13	
	III	1	3	

*PPF* Perforator propeller flap, *FF* Free flap, *SD* Standard deviation, *BMI* Body mass index, *DM* Diabetes mellitus, *PVD* Peripheral vascular disease, *HT* Hypertension, *ASA* American Society of Anesthesiologists

**Table 2:** Details of soft tissue defects

		**PPF**	**FF**	***p*** ** value**
Numbers of patients		15	18	
Numbers of flaps		18	20	
Etiology				** < ** ***0.001***
	Initial trauma	6	17	
	Postoperative wound necrosis / postoperative open wound	9	0	
	Postoperative infection	3	3	
Initial diagnosis				***0.04***
	Tibial shaft fx	5	9	
	Distal tibial fx	4	2	
	Ankle fx	5	0	
	Calcaneus fx	1	1	
	Talus fx	1	0	
	Lisfranc fx	0	4	
	Metatarsal fx	0	4	
	Traumatic ulcer	1	0	
	Achilles tendon rupture	1	0	
Open fracture (Gustilo classification)		5	16	***0.001***
	II	2	0	
	IIIA	1	0	
	IIIB	2	16	
Initial wound management				** < ** ***0.001***
	Other doctors	14	3	
	Reconstructive surgeons	4	17	
Open-wound periods (days): mean ± SD (range)^a^		21 ± 10 (0–42)	7 ± 7 (1–27)	** < ** ***0.001***
Location				***0.01***
	Middle 1/3	3	7	
	Distal 1/3	13	5	
	Heel	2	2	
	Mid-foot	0	6	
Defect size				** < ** ***0.001***
	Large (> 8 cm)	5	20	
	Middle (4–8 cm)	6	0	
	Small (< 4 cm)	7	0	
Internal fixation		15	14	0.40
	Intramedullary nail	5	6	
	Plate	7	7	
	External fixator	0	1	
	Screw or tension band	3	0	
Implant just under the defect		9	7	0.51

*PPF* Perforator propeller flap, *FF* Free flap, *fx* Fracture, *SD* Standard deviation

^a^Excludes cases with postoperative infection

### Flap details

Table 3 presents the specifics of the flaps used. Either the tibialis posterior artery (PTA) or the peroneal artery perforator was used for PPF; many flaps had a large rotation angle of 145 degrees on average. In the FF group, an anterolateral thigh (ALT) flap or latissimus dorsi myocutaneous (LD) flap was used. The PTA, tibialis anterior artery (ATA), or dorsalis pedis artery was used as the recipient vessel, and anastomosis was performed by ETS, except for the initial two surgeries. The flap size was significantly smaller in the PPF group compared to the FF group. In the PPF group, five cases of calcification of the major arteries were observed on radiographs. The operation time was significantly longer in the FF group, and the number of cases that required skin grafting to the donor was significantly higher in the PPF group.

**Table 3:** Flap details

		**PPF**	**FF**	***p*** ** value**
Numbers of patients		15	18	
Numbers of flaps		18	20	
Flap perforator origin	PTA	16	―	
	PA	2	―	
Rotation angle (degrees): mean ± SD (range)		149 ± 39 (45–180)	―	
Flap type	ALT	―	13	
	LD	―	7	
Recipient artery	PTA	―	11	
	ATA	―	3	
	Dorsalis pedis artery	―	6	
Arterial anastomotic type	End-to-end	―	2	
	End-to-side	―	18	
Flap size (cm^2^)		48 ± 17 (24–80)	118 ± 55 (40–202)	** < ** ***0.001***
Main artery calcification^a^		5	1	0.08
Operation time (min): mean ± SD (range)^b^		213 ± 71 (110–318)	605 ± 208 (361–1093)	** < ** ***0.001***
Skin graft for donor site		8	1	***0.007***

*PPF* Perforator propeller flap, *FF* Free flap, *PTA* Tibialis posterior artery, *PA* Peroneal artery, *SD* Standard deviation, *ALT* Anterolateral thigh flap, *LD* Latissimus dorsi myocutaneous flap, *ATA* Tibialis anterior artery

^a^Main artery from which the perforator branched used in PPF and anastomosed recipient artery using in FF

^b^Includes other procedures such as open reduction and internal fixation

### Flap outcomes and complications

The flap outcomes and complications in the two groups are presented in Table 4. The coverage failure rate was 22% in the PPF group and 5% in the FF group (OR, 0.18; 95% CI:0.02–1.83; *p* = 0.17). Surgical complications assessed according to the Clavien–Dindo classification did not differ between the two groups. Concerning flap-related early complications, the PPF group exhibited a significantly higher incidence compared to the FF group, particularly with venous congestion in 72% of the PPF group and 10% of the FF group (OR, 23.4; 95% CI: 3.91–139.92; *p* < 0.001). However, the complete necrosis rate was 11% in the PPF group and 5% in the FF group (OR, 2.38; 95% CI: 0.20–28.67; *p* = 0.59), with partial necrosis observed in 39% of the PPF group and 10% of the FF group (OR, 5.73; 95% CI: 1.00–32.67; *p* = 0.06), indicating no significant difference between the two groups. Concerning flap-related delayed complications, delayed osteomyelitis was not observed in the FF group, whereas in the PPF group, delayed osteomyelitis was observed in four cases (*p* = 0.04). In three out of four cases, reconstruction using free vascularized bone graft was needed.

**Table 4:** Flap outcomes and complications

		**PPF**	**FF**	***p*** ** value**
Numbers of patients		15	18	
Numbers of flaps		18	20	
Follow-up period (months): mean ± SD (range)		31 ± 24 (4–86)	24 ± 21 (3–64)	0.37
Coverage failure (rate)		4 (22%)	1 (5%)	0.17
Postoperative complications^a^	No complications	3	9	0.35
	Grade I	2	1	
	Grade II	1	0	
	Grade IIIa	4	3	
	Grade IIIb	8	7	
Flap-related early complications (< 3 weeks)		14	8	***0.03***
	Complete necrosis (rate)	2 (11%)	1 (5%)	0.59
	Partial necrosis (rate)	7 (39%)	2 (10%)	0.06
	Arterial thrombosis	0	1	1.00
	Venous congestion (rate)	13 (72%)	2 (10%)	** < ** ***0.001***
	Postoperative infection	2	7	0.13
Flap-related delayed complications (> 3 weeks)		5	1	0.08
	Delayed osteomyelitis	4	0	***0.04***
	Non-union	3	1^b^	0.22
Additional treatment until wound healing		14	12	0.31
	Secondary closure	5	4	0.71
	Debridement	3	5	0.70
	Implant removal or change	2	1	1.00
	Skin graft	1	3	0.61
	Leech therapy	2	0	0.22
	Secondary flap	4 (free ALT)	1 (free LD)	0.17
Additional surgery after wound healing		9	8	0.74
	Implant removal	5	2	0.39
	VBG for bone reconstruction	4	1	0.33
	Bone graft	0	3	0.10
	Arthrodesis	0	2	0.49
	Defatting	0^c^	1	1.00
	Tendon reconstruction	1	1	1.00
Patient mobility		0.4 ± 0.5	0.3 ± 0.6^d^	0.47

*PPF* Perforator propeller flap, *FF* Free flap, *SD* Standard deviation, *ALT* Anterolateral thigh flap, *LD* Latissimus dorsi myocutaneous flap, *VBG* Vascularized bone graft

^a^According to the Clavien–Dindo classification^21^

^b^Includes 1 pathological fracture

^c^Excludes cases involving defatting for secondary free flap

^d^Excludes 1 pathological fracture and 1 higher-order dysfunction

All cases of flap necrosis in both groups are detailed in Table 5. In the PPF group, complete flap necrosis was observed in two flaps (flaps numbered 4 and 13): one due to postoperative infection in the distal third of the lower leg, and the other associated with a soft tissue defect from an open fracture in the middle third of the lower leg. Both patients were in their 20s and had no comorbidities, but postoperative ischemia occurred, and despite attempts to reverse flap rotation, the blood circulation did not resume. Among the seven flaps from six patients with partial necrosis in the PPF group, five flaps from four patients aged 60 years or older, four flaps from three patients with diabetes mellitus, and one flap from a patient undergoing hemodialysis was included. Partial necrosis was observed in all five flaps, with calcification present in the main arteries bifurcating the perforator.

**Table 5:** Cases of complete and partial flap necrosis

Group (flap No.)	Age/Sex	Comorbidity	Initial trauma (location)	Flap size (cm^2^)	Perforator origin (rotation angle)	Recipient artery	Complication	Additional surgery for coverage	Additional reconstruction
PPF(4)	22 M	none	talus fx. (distal 1/3)	30	PTA (180)	―	complete necrosis non-union	secondary free flap (ALT)	VBG (VSG)
PPF(5)	57 M	DM, HT, HD smorking	ankle fx. (distal 1/3)	59	PTA^a^ (160)	―	partial necrosis	secondary free flap (ALT)	―
PPF(7)	95 F	breast & lung Ca	distal tibial fx. (distal 1/3)	24	PTA (120)	―	partial necrosis	implant removal secondary closure	―
PPF(11)	61 F	DM, liver cirrhosis	calcaneus fx. (heel)	63	PTA^a^ (180)	―	partial necrosis	debridement	―
PPF(12)	47 M	HT smorking	tibial shaft fx. (distal 1/3)	80	PTA (180)	―	partial necrosis	secondary closure	―
PPF(13)	27 M	Depression smorking	tibial shaft fx. (middle 1/3)	41	PTA (170)	―	complete necrosis delayed osteomyelitis	secondary free flap (ALT)	―
PPF(15)	82 F	endomaterial Ca, angina pectoris	ankle fx. (distal 1/3)	64	PTA^a^ (170)	―	partial necrosis	secondary closure	―
PPF(17)	71 F	DM, HT	ankle fx. (distal 1/3)	42	PTA^a^ (170)	―	partial necrosis delayed osteomyelitis	implant removal debridement	VBG (VSG)
PPF(18)				62	PA^a^ (180)		partial necrosis	secondary free flap (ALT)	―
FF(2)	63 M	HT smorking	Lisfranc fx. (distal 1/3)	101	―	ATA	complete necrosis	secondary free flap (LD)	―
FF(3)	73 M	DM, HT smorking	distal tibial fx. (distal 1/3)	87	―	PTA	partial necrosis	skin graft	―
FF(4)	54 F	none	tibial shaft fx. (middle 1/3)	197	―	PTA	partial necrosis	debridement	―

*PPF* Perforator propeller flap, *fx* Fracture, *PTA* Tibialis posterior artery, *ALT* Anterolateral thigh flap, *VBG* Vascularized bone graft,*VSG* Vascularized scapular bone graft, *DM* Diabetes mellitus, *FF* Free flap, *HT* Hyper tension, *HD* Haemodialysis *Ca* Cancer, *PA* Peroneal artery, *ATA* Tibialis anterior artery, *LD* Latissimus dorsi myocutaneous flap

^a^Vascular calcification

In the FF group, complete necrosis developed in one ALT flap where ETE anastomosis was performed using the zone-of-injury artery selected as the recipient vessel (flap numbered 2). Additionally, two flaps in the FF group experienced partial necrosis: one LD flap exhibited marginal necrosis, likely due to excessive peeling of the skin flap from the muscle, while the other ALT flap showed partial necrosis attributed to excessive defatting.

**Fig. 2:**
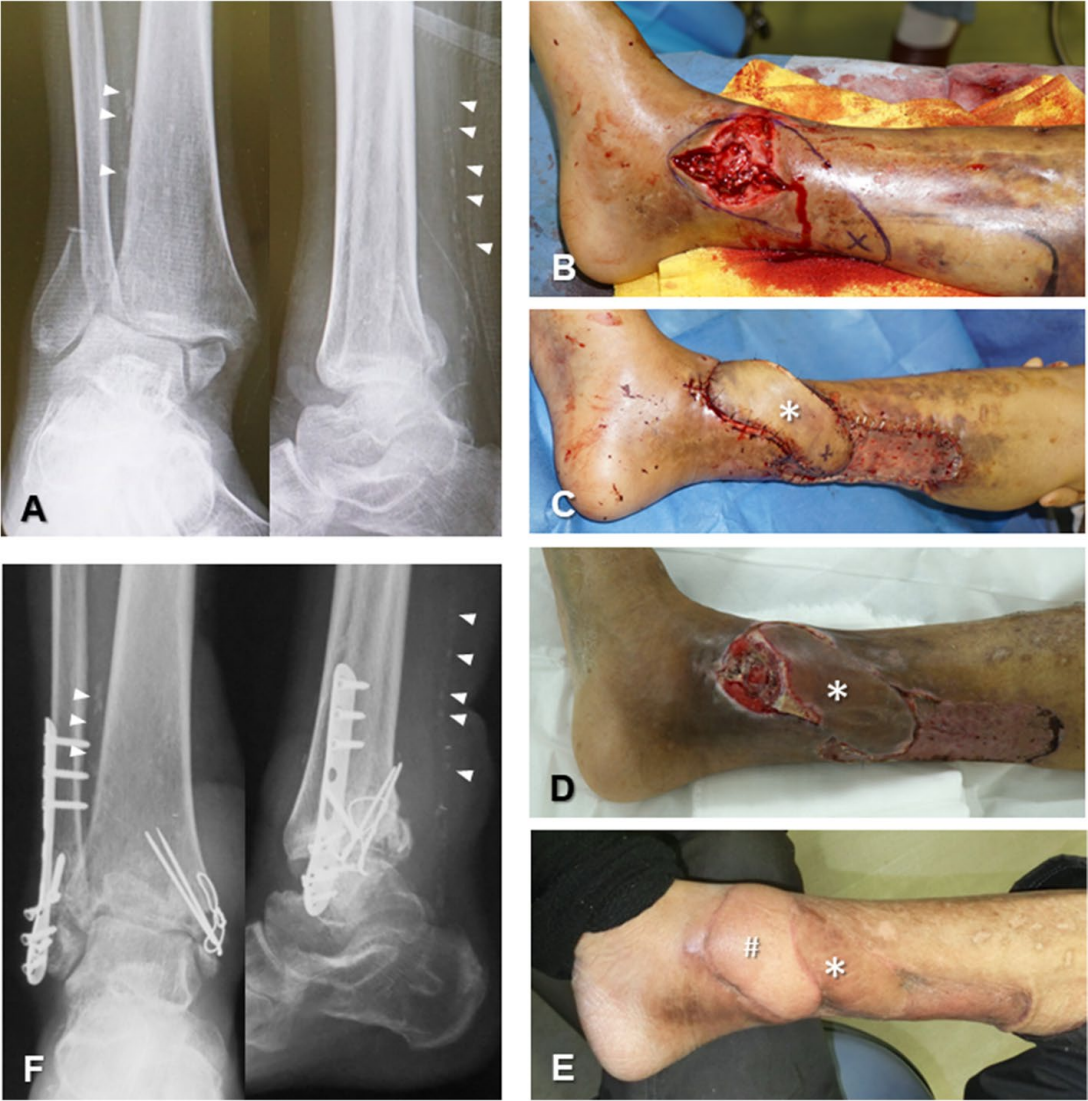
**A**, **B** A 57-year-old man (case number 5 in PPF group) on haemodialysis with an ankle dislocation fracture with soft tissue necrosis. Simultaneous osteosynthesis and soft tissue reconstruction was planned. **C** The perforator-based propeller flap (asterisk) using the posterior tibial artery perforator was elevated, and a split-thickness skin graft was inserted at the donor site. **D** Congestive partial necrosis at the tip of the flap and exposure of the implant at the medial malleolus were observed. Twenty-five days after insertion of the propeller flap, soft tissue reconstruction using an anterolateral thigh free flap was performed using the tibialis posterior artery as the recipient vessel. **E** The findings at 6 months after surgery. The soft tissue defect was completely covered with the anterolateral thigh free flap (hashmark). **F** X-rays at 6 months after surgery showed bone union. Calcification (arrowhead) can be seen along the course of both ATA and PTA

**Fig. 3:**
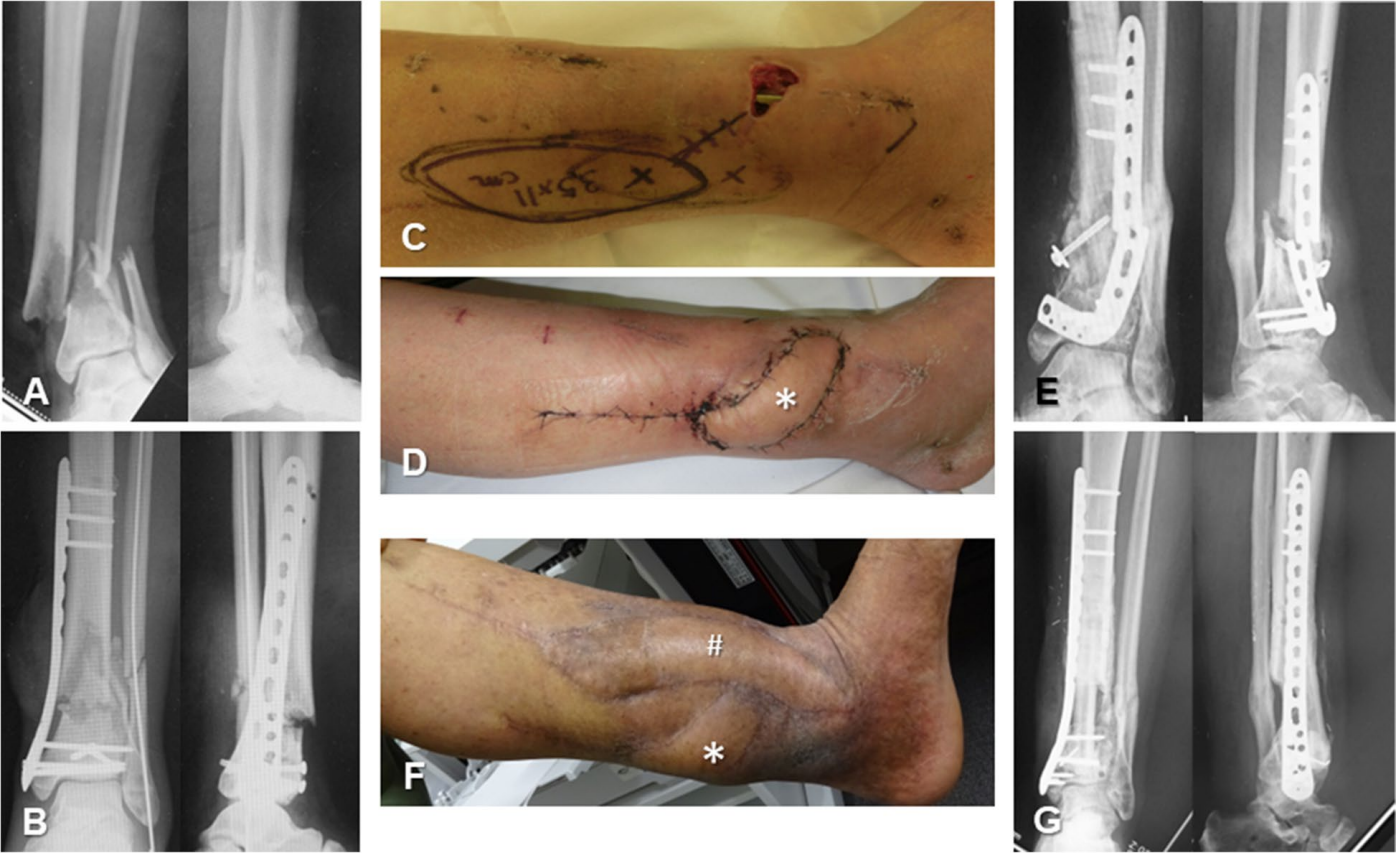
**A** A-65-year-old man with an open distal tibial fracture. **B**,** C** After osteosynthesis with an anterolateral plate, a 3 × 3 cm soft tissue defect with plate exposure remained on the anterior surface of the tibia. **D** Three weeks after osteosynthesis, soft tissue reconstruction was performed using a tibialis posterior perforator-based propeller flap (asterisk). The flap survived intact without complications such as congestion, but osteomyelitis occurred at 5 months after insertion of the propeller flap. Debridement and re-osteosynthesis with an autogenous iliac bone graft were performed. **E** X-rays 1 year after re-osteosynthesis. Implant breakage caused by infectious pseudoarthrosis was confirmed. **F** Bone reconstruction was performed using a contralateral free vascularized fibula bone graft (hashmark). **G** X-rays 2 years after insertion of the vascularized bone graft showed complete bone union

Among the flaps affected by necrosis, four were in the PPF group (flaps numbered 4, 5, 13, and 18) (Fig. 2), and one in the FF group (flap numbered 2) required further reconstruction with an FF due to implant/fracture exposure resulting from necrosis. In the FF group, seven flaps developed early postoperative infection, which resolved within a few days following debridement under the flap. Delayed osteomyelitis occurred after soft tissue healing in four flaps in the PPF group (one at 2 months, two at 5 months, and one at 7 months after flap insertion), and pseudoarthrosis was observed in three flaps. Four patients in the PPF group and one patient in the FF group underwent vascularized bone reconstruction following soft tissue healing (Fig. 3). The requirement for additional surgeries for defatting and/or implant removal, as well as the final walking function, did not differ significantly between the groups.

## Discussion

Considering the variability in outcomes based on surgical proficiency and the nature of soft tissue defects, assessing the effectiveness of PPF and FF for trauma-related lower leg and foot soft tissue defects remains challenging. This retrospective study faces limitations, notably the disparity in soft tissue defect backgrounds between the two groups. Nonetheless, it marks the inaugural comparison of PPF and FF for such trauma-related defects performed at the same facility. While no significant differences were observed in coverage failure rates, complete flap necrosis, or partial flap necrosis between the groups, four PPF cases required FF reconstruction due to implant/fracture exposure from necrosis, compared to one in the FF group. Furthermore, four PPF cases developed delayed osteomyelitis post-healing, with three necessitating reconstruction using free vascularized bone grafts. Although studies have reported that PPF is a simpler, cosmetic, and less invasive reconstruction procedure than FF [12, 15, 17, 18, 23], surgeons should not underestimate the risk of serious complications such as flap necrosis and delayed osteomyelitis when PPF is applied for traumatic reconstruction of the lower leg and foot.

In lower leg soft tissue reconstruction, pedicled flaps are considered to have a higher risk of partial flap necrosis compared to FFs [24]. Bekara et al. [23] compared the incidence of partial necrosis in PPF and FF groups for lower leg soft tissue reconstruction, reporting a complication rate of 6.88% in the PPF group compared to 2.70% in the FF group, which was significantly higher. Unlike soft tissue defects following tumor resection, those associated with lower leg trauma often involve trauma extending to the surrounding areas of the defect. Consequently, pedicled flaps based on the surrounding defect tissue are believed to have a higher risk of flap necrosis compared to FFs harvested from healthy tissue [25]. Reports on PPF for lower leg skin defects related to trauma often indicate a higher incidence of flap necrosis compared to non-traumatic cases [13, 16, 26], with Vathulya et al. [8] specifically noting a higher complication rate of flap necrosis among pedicled flaps. In our PPF outcomes, the rates of partial necrosis and complete necrosis were 39% and 11%, respectively, mirroring previous reports. Therefore, when considering PPF for lower leg and foot soft tissue reconstruction following trauma, careful patient selection is warranted compared to conventional pedicled flaps and FFs.

Even if necrosis occurs after PPF surgery, most cases can be managed with minor procedures such as conservative treatment or skin grafting, and few complications require major revision surgery [23, 27]. However, in trauma-related soft tissue reconstruction, partial necrosis can result in implant/fracture exposure, necessitating urgent secondary flap reconstruction in certain cases. Guiller et al. [10] reported partial necrosis or dehiscence in 10 of 21 (48%) flaps, with secondary FF required in three of 21 (14%) cases involving PPF for trauma-induced soft tissue defects. In our cases, two of seven (29%) flaps with partial necrosis required reconstruction with FFs due to exposure of implants or fracture sites. It is important to recognize in trauma reconstruction that partial flap necrosis can lead to serious complications, differing from those in non-traumatic cases.

Age ≥ 60 years and the comorbidity of diabetes mellitus or peripheral vascular disease are considered risk factors for PPF failure [16, 23]. In this study, these risk factors were associated with most flaps exhibiting partial necrosis after PPF. Special attention should be paid to patients with calcification of the main arteries bifurcating a perforator. In addition, some soft tissue defects were caused by wound dehiscence after osteosynthesis, and such patients are likely to have low wound healing ability. Although PPF is thought to be a simple procedure with a short operation time and low risk of systemic complications [28], the possibility of poor blood flow in the flap should be considered in patients with serious comorbidities [16, 29].

Delayed osteomyelitis in the lower leg and foot is identified as a serious complication necessitating complex long-term treatment [30, 31]. Notably, no instances of osteomyelitis were observed in the FF group; however, 20% of patients who underwent PPF experienced delayed osteomyelitis following soft tissue coverage, with three flaps ultimately requiring vascularized bone graft reconstruction. Two potential reasons may account for the occurrence of delayed osteomyelitis in the PPF group. Firstly, the PPF group exhibited a higher proportion of cases referred midway through initial treatment by other physicians for soft tissue defect reconstruction, compared to the FF group, raising the possibility of inadequate initial debridement. Moreover, the duration from wound exposure to reconstruction was significantly prolonged in the PPF group compared to the FF group, potentially facilitating bacterial invasion and establishment of infection within the long-term open wound. In the context of posttraumatic soft tissue reconstruction, effective infection control hinges on thorough debridement of open wounds and administration of appropriate antibiotics prior to soft tissue reconstruction. Secondly, a substantial discrepancy in flap blood flow volume between the two groups may contribute to the development of delayed osteomyelitis. While the FF group features myocutaneous flaps, characterized by robust vascularization and the ability to fill three-dimensional soft tissue defect wounds, thereby aiding infection control, the PPF group may experience instances of partial necrosis attributed to the flap's reliance on marginal blood flow for survival.

Although the usefulness of FF for trauma reconstruction of the lower leg is widely recognized [1, 2, 6], caution is required to avoid inducing vascular spasm in the zone of injury [32, 33]. In this study, total necrosis of the skin flap occurred due to ETE anastomosis using zone-of-injury blood vessels as the recipient. ETS is less likely to cause vascular spasm and may be a safer anastomosis method for reconstruction after trauma [26, 34]. In this case, it is considered possible to avoid complications of irreparable vascular spasm by performing an anastomosis using ETS with a spared PTA as the recipient.

Some surgeons have emphasized the significance of experience in achieving success with the PPF technique in reconstructing lower leg soft tissue defects [13, 16, 35]. However, even experienced surgeons experience flap failure [35], and some have become cautious about applying PPF for traumatic soft tissue defects and have reverted to FF [10, 16]. In this study, all FFs were able to cover the defects except for one flap early in the time frame of this study, but the risk of flap failure decreased as the surgeons gained more experience. The failure of PPF coverage was equally observed in all the stages. When PPF is unsuccessful in the reconstruction of injuries related to trauma, more difficult reconstruction requiring microsurgery may be necessary and may include super drainage for venous congestion, secondary FF for flap failure, or reconstruction using a vascularized bone graft for delayed osteomyelitis [10, 16]. We emphasize that PPF is not a simple non-microsurgical procedure for reconstructing lower leg trauma defects in patients with poor physical condition.

While PPF presents certain risks, it undeniably serves as a valuable method [3, 12, 13, 17, 18]. To safely apply PPF to lower leg trauma, one must consider various factors beyond patient background and the local conditions of the open wound site. This includes examining the circumstances surrounding the wound, such as the energy involved at the time of injury, initial diagnosis, fracture type, Gustilo classification, initial wound management status, and duration of wound openness, among others [16, 23]. Surgical techniques should be executed with care, with particular attention paid to pedicle dissection and rotation [22]. Although evaluating angiosomes post-trauma poses challenges [16], reports recognize the ability to objectively assess skin flap viability using intraoperative indocyanine green [28, 36]. When considering PPF for trauma, a more cautious approach and technique are necessary compared to FFs to prevent serious complications post-surgery. Being prepared to address reconstruction with FFs in case of flap necrosis is considered crucial to avoid severe complications.

Our study had several limitations. Firstly, it was retrospective in nature, and the sample size in both groups was small. Additionally, as mentioned earlier, the backgrounds of the two groups were not perfectly matched. Secondly, we acknowledge the possibility of bias in the selection of treatment between the two modalities. Larger sample sizes and more rigorous study designs will be necessary for future research to safely advocate for the use of PPF in trauma-related soft tissue reconstruction of the lower leg and foot.

## Conclusions

In our study, we compared the outcomes of PPF and FF procedures performed for the repair of trauma-related soft tissue defects in the lower leg and foot. When utilizing PPF for lower leg trauma reconstruction, surgeons must consider the extent of the zone of injury and the potential for flap complications, including venous congestion and partial or complete necrosis, which could compromise limb salvage. Conversely, FF offers a solution by transferring well-vascularized and uninjured tissue to cover the defect, relying on a vascular supply from healthy sections of the vascular tree outside the zone of injury.

## Data Availability

The datasets generated during and/or analyzed during the current study are available from the corresponding author on reasonable request.

## Abbreviations

FF: Free flap
PPF: Perforator-based propeller flap
ASA: American Society of Anesthesiologists
ETE: End-to-end
ETS: End-to-side
OD: Odds ratios
CI: Confidence intervals
PTA: Tibialis posterior artery
ALT: Anterolateral thigh
LD: Latissimus dorsi myocutaneous
ATA: Tibialis anterior artery
